# Supplementary figures and images for: RNA Sequencing Reveals a Strong Predominance of THRA Splicing Isoform 2 in the Developing and Adult Human Brain
Source: Int J Mol Sci. 2024 Sep 13;25(18):9883. doi: 10.3390/ijms25189883 (PMC11432079; doi:10.3390/ijms25189883)

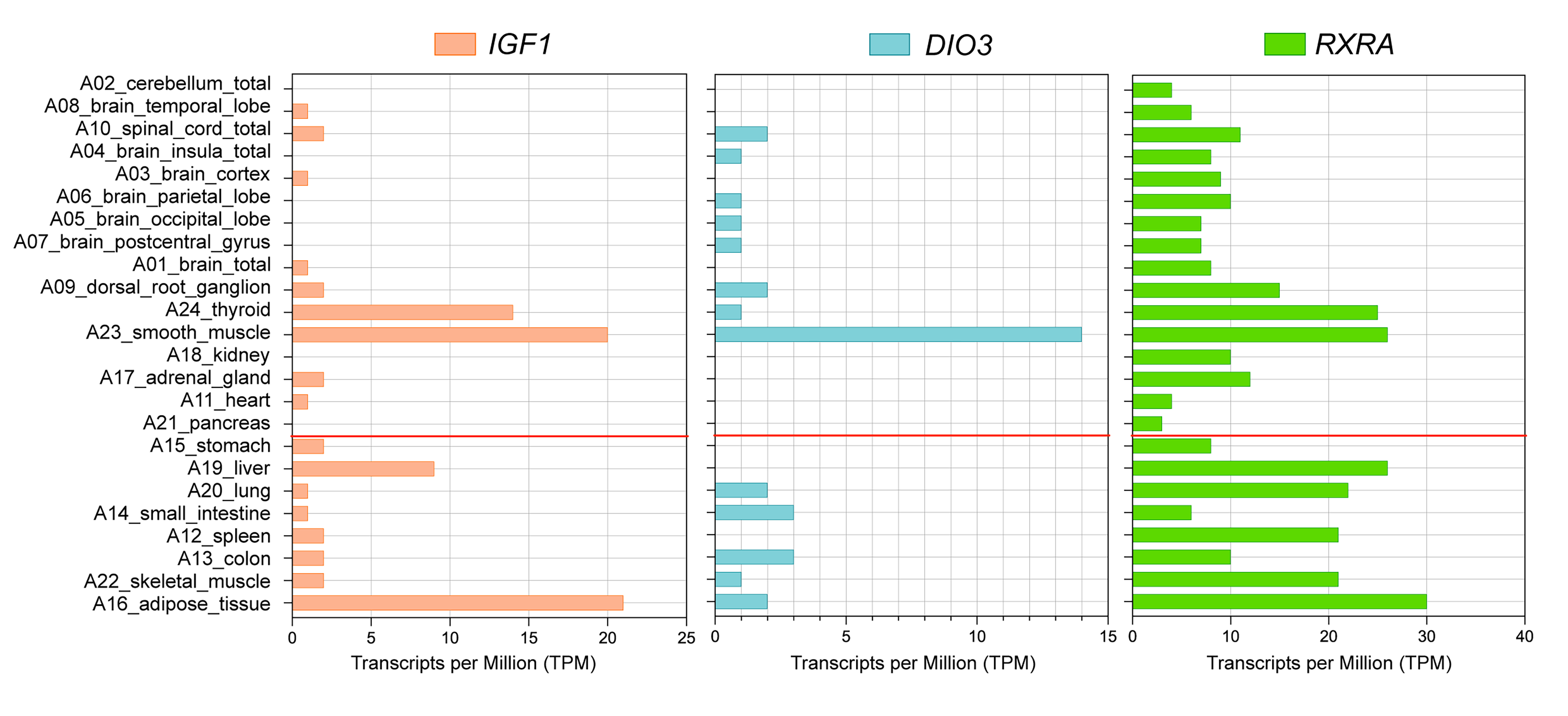

Supplement: Supplementary file 1 [file ijms-25-09883-s001.zip › Fig_S1.tif]
